# How to optimise the use of innovative invasive devices: considerations for feasibility studies

**DOI:** 10.1186/s40814-026-01797-8

**Published:** 2026-03-10

**Authors:** Emily Hotton, Julia Wade, Daisy Elliott, Erik Lenguerrand, Joanna Crofts, Natalie Blencowe

**Affiliations:** 1 Women and Children’s Research Centre, Southmead Hospital, North Bristol NHS Trust, Bristol, UK; 2 Translational Health Sciences, University of Bristol, Southmead Hospital, Bristol, UK; 3 NIHR Bristol Biomedical Research Centre, University Hospitals Bristol and Weston NHS Foundation Trust, Bristol, UK; 4 Centre for Surgical Research, Populational Health Sciences, Bristol Medical School, University of Bristol, Bristol, UK

**Keywords:** Assisted vaginal birth, Feasibility, Intrapartum research, Medical device, Methodology, OdonAssist, Safety

## Abstract

Innovation in medical devices is essential for advancing patient care but has been undermined by inadequate evaluation and regulation. The UK vaginal mesh scandal exposed systemic flaws in medical device evaluation, where insufficient pre-market testing and ineffective post-market surveillance caused harm to countless women. Unlike pharmaceutical products, medical devices often enter clinical practice without robust evidence or long-term safety data. However, evaluation of devices can present methodological challenges, particularly when they are used during invasive procedures, given their multifaceted nature and contextual variables. Using the ASSIST study and the OdonAssist™ inflatable device for assisted vaginal birth as a case study, we wish to highlight these challenges and present a model for conducting feasibility studies of complex interventions. Early feasibility studies of the OdonAssist device demonstrated that although women accepted the innovation, the success rate of assisted vaginal birth was lower than anticipated. An embedded qualitative evaluation provided critical insights, revealing key factors influencing device use, design, and operator technique. The use of qualitative case study methodology allowed for systematic observation, stakeholder feedback, and iterative device refinement. For example, issues such as accidental activation of the deflation button were identified, leading to real-time design modifications in conjunction with industry partners. Stakeholder engagement—including patients, clinicians (doctors and midwives), ethics committees, and industry partners—was integral to optimising study design, device functionality, and training programmes. This collaborative, data-driven approach expedited the identification of modifications to device use, necessitating design changes and thereby ensuring device safety and usability. The evaluation of innovative devices like the OdonAssist requires a holistic approach that incorporates qualitative methodologies, stakeholder collaboration, and iterative feedback. Embedding case study research within feasibility trials ensures rapid identification of challenges, supports timely modifications, and fosters safer device implementation. Future research should explore video-recorded observations and enhanced patient feedback to refine device technique and training further.

## Background

**Fig. 1:**
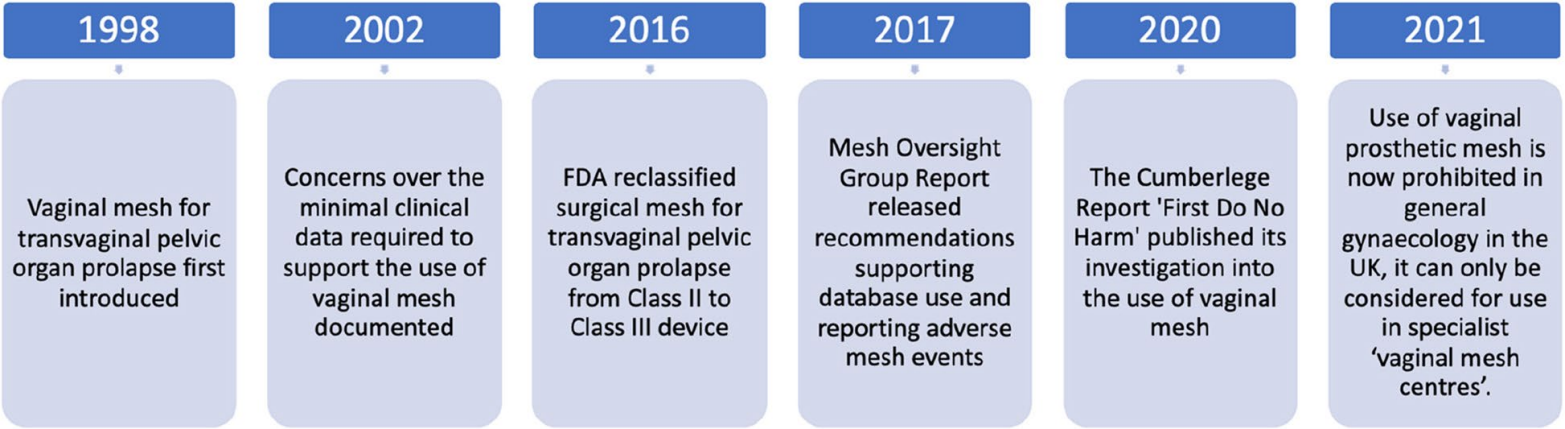
Timeline of the vaginal mesh controversy [3, 5, 6]. A Class II medical device refers to mesh products initially cleared through the approval process as moderate risk devices, whereas a Class III medical device designation (to which vaginal mesh for pelvic organ prolapse was later reclassified) identifies it as high-risk, requiring rigorous pre-market approval due to potential serious complications

Innovation is critical to advancing and improving patient care, yet the evaluation of novel invasive procedures and devices is often haphazard, with several recent high-profile controversies [1–3]. The UK vaginal mesh scandal highlights a troubling systemic failure in the evaluation and regulation of innovative medical devices [3]. Synthetic polypropylene prosthetic mesh was widely adopted for treating women with vaginal prolapse or urinary stress incontinence without sufficient evidence from randomised controlled trials or long-term safety evaluation. This caused severe harm to many women including chronic pain, infection, organ perforation, and mesh erosion (Fig. 1) [4]. These outcomes were devastating, often leaving women with lifelong physical and emotional morbidity. Before agreeing to surgery, women were unaware of the significant risks associated with mesh use, as clinicians themselves lacked comprehensive evidence to fully inform them.

Until recently, medical devices such as vaginal mesh were allowed onto the market with minimal pre-market testing and evaluation, in stark contrast to the stringent requirements for pharmaceutical products. Post-market surveillance was similarly deficient, with reports of adverse outcomes failing to trigger timely and decisive action. This regulatory laxity allowed devices to be widely used before their long-term safety and effectiveness were properly understood. The vaginal mesh controversy prompted a suspension of mesh procedures in 2018, a review of surgical practices, and thousands of compensation claims. Use of vaginal mesh in the UK is now prohibited outside of a limited number of specialist ‘vaginal mesh centres’ [3].

To mitigate against future harm, an independent report was commissioned entitled ‘First Do No Harm’ [3], recommending mandatory, robust evaluation of novel procedures and devices to prevent their introduction into the global market without reliable evidence, as well as mandatory registries for all implantable devices. However, there has been increasing recognition that evaluating the use of innovative invasive procedures and devices presents a unique set of methodological challenges [7, 8]. A major issue is that invasive procedures and/or devices are considered to be complex interventions, comprising multiple interacting components that may act independently or interdependently to influence outcomes [9]. Unlike pharmacological interventions (where there is a prescribed dose, route, timing, and duration), complex interventions may be difficult to define. This is because, in addition to the procedure itself (which comprises numerous components and steps), there are multiple accompanying co-interventions occurring before, during, and after the procedure, together with various contexts, processes, people, and decisions [9, 10]. This complexity may be even greater when an invasive procedure or device is relatively novel, because the device itself may undergo further development.

The Medical Research Council guidance recommends the use of feasibility studies in the development and evaluation of complex interventions to assess the practicality of proceeding to a full trial. It suggests that feasibility studies are used to (i) assess acceptability and implementation, (ii) test procedures and protocols, and (iii) evaluate key parameters [7]. Other benefits to feasibility work include documentation of procedural refinements and recognition of uncertainties surrounding device use. However, optimal ways of achieving this are uncertain and little is known about the most appropriate and robust way to conduct feasibility phase evaluations of novel invasive procedures to inform main phase clinical trials of effectiveness.

This paper demonstrates a methodological approach for exploring complex interventions, using the ASSIST study and OdonAssist™ device [11] as a case study. We detail crucial methodological considerations for gathering information about novel invasive procedures and devices in a systematic way during feasibility studies and prior to any randomised evaluation of effectiveness.

## The OdonAssist™ device

**Fig. 2:**
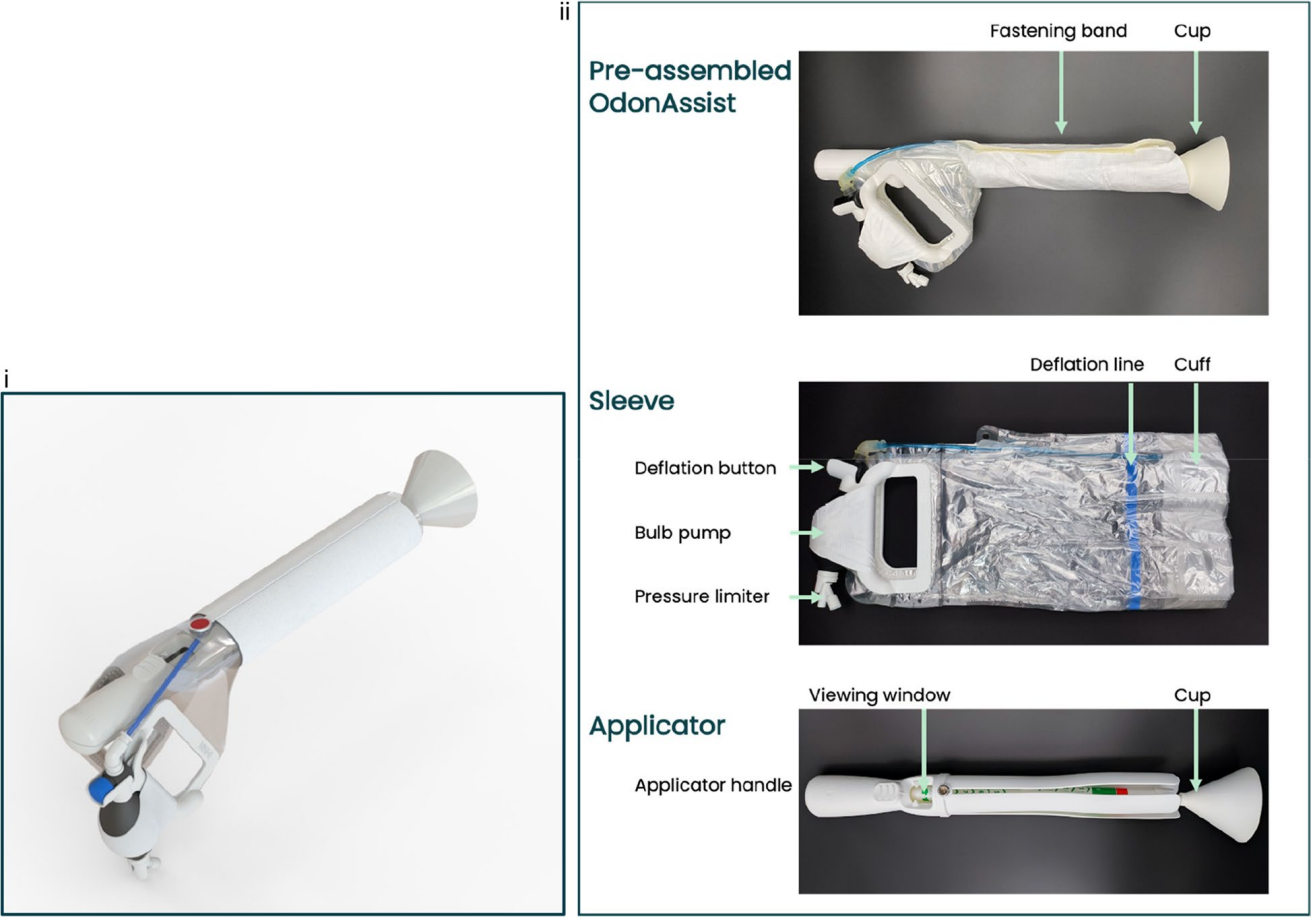
The OdonAssist device: (**i**) assembled device and (**ii**) component parts (sleeve and applicator). ©2023 Maternal Newborn Health Innovations, PBC. Permission for publication granted by Maternal Newborn Health Innovations, PBC

The OdonAssist is a novel device for assisted vaginal birth. Currently, two devices are available in the UK for assisted birth: ventouse and forceps. Both require a high level of operator training and skill as they are associated with potentially significant maternal and fetal morbidity [12]. This morbidity has prompted a global decline in assisted vaginal birth and an increase in caesarean births [13, 14]. The OdonAssist device was therefore designed with the aim of being easier and safer to use, and thus potentially increasing worldwide access to assisted vaginal birth (Fig. 2). The device has a novel mechanism of action via a positive pressure circumferential air cuff that is inflated over the fetal head, providing flexion and traction for assisted birth [11].

## OdonAssist research

### Prior pre-clinical and clinical research

The OdonAssist device was first studied in South America and Argentinian women who were about to achieve a spontaneous vaginal birth (i.e. did not require assistance). Results from this study suggested it was feasible to apply the device to the fetal head in the final stages of labour, and there were no concerning maternal or fetal complications [15]. Subsequently, between 2017 and 2019, pre-clinical testing was undertaken to explore perineal distension, fetal head pressures, human factors such as device design and device use, as well as safety and functionality [16–19]. This enabled development of the initial Instructions for Use (IFU), a legal document required for any medical device which also forms the foundation of its training programme by providing essential guidelines on correct device operation, safety precautions, and clinical parameters.

### The first feasibility study of the OdonAssist (the ASSIST study)

The first feasibility study of the OdonAssist was undertaken in the UK and was the first time the device was used in women who required an assisted vaginal birth at the end of their labour [22]. Women were recruited using a bespoke study information video developed by the study team and approved for use by the ethics committee alongside the paper Patient Information Leaflet. The ASSIST study was initially designed to be a purely quantitative study, but was expanded to include an embedded qualitative evaluation with an aim to more effectively understand optimal device use [20]. The 78% study recruitment rate indicated that women found the device acceptable [20, 21]. However, although there were no documented significant adverse reactions to the device use at birth, the success rate (as measured by vaginal birth using the OdonAssist) was lower than expected at 48% [20, 22]. The embedded qualitative evaluation, stakeholder engagement, and close collaboration with industry were critical in delivering valuable insights into how the device was used and facilitating systematic evaluation of the device and its use [23]. Each of these will be considered in turn.

## Methodological considerations

### Embedded qualitative research

Qualitative case study methodology [23] embedded within the ASSIST study enabled rapid triangulation of insights from multiple data sources. Case studies comprised data from at least one of the following sources for each attempted OdonAssist birth: in-person researcher observations of the AVBs and face-to-face interviews with women, midwives, and operators. The aim was to investigate whether use of the novel device could be optimised in a real-life clinical setting, with a specific focus on (i) observing how the OdonAssist was used and how use varied with contextual factors (e.g. fetal position or analgesia used); (ii) detailing the ‘usual’ steps for use, optimal device design and the clinical parameters for device use; (iii) exploring how operators experienced the device in the clinical setting and how effective they found simulation training to be; and (iv) exploring views and experiences regarding the standardisation of medical device use and adherence to device use within trials [8].

#### Participants’ experiences of the novel device

Semi-structured interviews explored women’s experiences of the OdonAssist and their participation in intrapartum research [23]. Women valued the multimedia information provision during recruitment and reported that in-person observation of their assisted vaginal birth was acceptable, supporting use of observation in novel device feasibility studies [8].

#### Optimisation of device use: device design, technique, and clinical parameters

Participant observation and semi-structured interview data with device operators and midwives were analysed to explore the introduction of OdonAssist in detail. Data from these sources were crucial in further developing the IFU during the study: the IFU was considered a ‘live document’ that was continually reviewed and adapted. It is recommended that the technique for the device should be modified as data were gathered during clinical evaluation [24, 25], and this model was followed in the ASSIST study. This approach to technique adaptation can be hard to achieve, but the qualitative analysis helped us achieve this. The IFU was written by the manufacturer before the study started and supported our evaluation by presenting a starting point and transparent guideline for use, which were then systematically reviewed, updated, and refined. These updates were based on observations and interviews supplying real-time suggestions. Discussions between operators, engineers, and industry were held to determine which suggestions should be brought formally into the IFU. Any device training programme is designed around the IFU to ensure healthcare professionals understand the procedural steps, adhere to standardised practices, and reduce variability in clinical outcomes. Updates to the IFU are reflected in ongoing training to ensure users are always informed of the latest device specifications. Case study methodology enabled behaviours observed during the assisted births to be investigated further during interviews. Operators had the opportunity to quantitatively self-report their own observations of an assisted birth using case report forms and Likert scales. However, crucial aspects were not captured via this method and were only captured due to embedded case study methodology. For example, twice the accidental pressing of the deflation button was not noted by the operator, but reported by the observer. All operators were subsequently informed of this risk and ultimately a design change in the device was agreed. Device amendment requests were discussed with design engineers in real-time, enabling them to work on design iterations whilst the study was still running.

Systematic triangulation of data during analysis [26] also allowed for rapid feedback of insights into device use as these emerged to operators. When exploring the clinical parameters for use, each birth was plotted on a timeline of cases and with failures scrutinised specifically to explore commonalities in fetal presentations that were causing issues. We were then able to have targeted discussions about these in interviews with operators. We recommend use of case study methodology whenever innovative devices are introduced to clinical trials and settings. Its use allows for rapid and efficient assessment of device design and use and can support timely iterative adaptations to the device or its use to optimise both.

### Stakeholder engagement

The study involved multiple stakeholders, including women participating in the feasibility trial, clinicians (obstetricians and midwives), researchers, ethics committee members, and industry partners. Participants provided input on both study methods and outcomes through patient and public involvement (PPI) sessions that informed recruitment materials, consent processes, and study design. Women’s feedback, gathered via semi-structured interviews and observations, influenced how the device was introduced, how data were collected, and what outcomes were prioritised, such as acceptability, usability, and safety. Clinicians contributed insights that shaped the IFU, operator training, and refinements to device technique. This ongoing, collaborative input ensured the study methods, procedures, and outcomes reflected real-world clinical practice and patient experience.

#### The impact of patient and public involvement

Researchers make ‘gate keeping’ assumptions all the time [27], but with patient and public involvement, researchers can make modifications in line with what will benefit the target population. Our concern at the outset of the feasibility study was that women would not want their births observed during the evaluation of an innovative device. Contrary to our assumptions, women expected and accepted that their births be observed, believing that it was the only way the research team could understand the device use and thus improve intrapartum care for other women [23]. Patient and public involvement was crucial to the development of participant facing materials and the protocol, with the former then well received by participants.

A crucial step for any study is dissemination (of positive and negative findings) to academic colleagues and the public—this importantly should involve the participants [28]. After data collection was complete in the feasibility study, we invited all participants, their babies, and families to attend a ‘tea party’. The aim was to provide participants with a written lay summary of findings, a certificate of participation for them and their babies, and a small presentation about the next steps in the development of the OdonAssist. Participants who did not want or were unable to attend were provided the same information by post. Participants valued the prompt and honest feedback regarding the study, and the research staff and operators enjoyed the opportunity to reconnect with participants. The NIHR CRN West of England came to the event as it was considered an exemplar for participant dissemination https://www.youtube.com/watch?v=mioXSqZr3sg. We recommend actively involving participants throughout the lifecycle of the research. The findings from this research were subsequently shared at international obstetrics and gynaecology meetings, where they generated significant interest in methodological approaches to device evaluation, and several related papers have been published in peer-reviewed journals to disseminate these insights more widely [8, 20, 22, 23].

#### Role of the ethics committee

Study design is the most vital element to a trial; it lays the foundation for the clinical research. RCTs evaluating innovative invasive devices can be challenging as there are many variables to consider such as the patient, device, operator, setting, co-interventions, and associated health care professionals, especially in emergency settings such as this. Close liaison with the ethics committee before submission enables questions and queries to be pre-emptively addressed. The study team contacted the chair of the committee to ask specific questions related to participant recruitment and exclusion criteria that had been challenging to decide during the creation of the protocol. Using the expertise of the committee in this way strengthened our study design and established an extremely positive relationship with our ethics committee. Having a committee with previous medical device experience and discussing ethical queries before submission of study documents for approval opened dialogue to guarantee that both parties understood the aim of the research, enabling us to work collaboratively to optimise study design for patients and researchers, including timing of recruitment (antenatally and intrapartum), provision of recruitment information, and neonatal outcomes.

### Collaboration with industry

We recommend having industry and/or device manufacturers as part of the extended team. Industry requires clinical research to develop and ultimately sell their products, and researchers and manufacturers have a shared goal of improving patient care. Engagement with the manufacturer needs to start from the moment the protocol is being drafted. They have no influence over study design, but keeping them informed from the beginning starts the relationship on a good footing. It is crucial to identify key personnel with whom to liaise and be aware you may need different people at different times of the trial.

#### Pre-study

We liaised regularly with the device manufacturers before the study started to ensure that key device outcomes were captured in the protocol. Procedures for device use during the study (Instructions for Use) and inspection post-use needed to be agreed a priori.

#### During the study

We had regular meetings to discuss use of the device, aspects of device use that operators felt were challenging or positive and feedback from women. When suggestions for device alterations became apparent to the clinical research team, we then had a clear communication channel by which to relay these swiftly to the manufacturers. They in turn started in-house development and testing, meaning there was minimal delay in providing the clinical studies with a modified device [8].

#### After the study

The device manufacturers have been actively engaged with the clinical teams not only in disseminating but also in helping develop a device training programme, clinical implementation packages, and discussing strategies for definitive studies and post-market surveillance.

#### Involving industry in key meetings regarding the understanding of device use in clinical trials

Device design cannot be considered in isolation*.* Device technique is inextricably linked with device design and clinical parameters for device use. Altering one is likely to impact on the others. Without this detail, trialists and clinicians will be unable to replicate device use in clinical practice in the same way it has been implemented during the evaluation. This can be demonstrated in the development of the OdonAssist. The first-in-human study of the device, led by the World Health Organization between 2011 and 2017, used four different device prototypes [15] with investigators stating that each modification did not interfere with the study results. This statement is hard to accept as it is preceded by a statement stating that the design changes improved usability, aided insertion, and ensured correct cuff pressure [15]. Furthermore, there are no details as to how many births were assisted by each iteration of the device and no comparison of success rates between iterations.

By having a relationship with industry that enabled us as researchers to voice our opinions/views surrounding the use of the device to a receptive team, we were able to suggest modifications to device design that were ultimately adopted and incorporated into the devices used in the feasibility studies, with documentation of adaptations maintained throughout [22].

### Framework for evaluating device use in feasibility studies

**Fig. 3:**
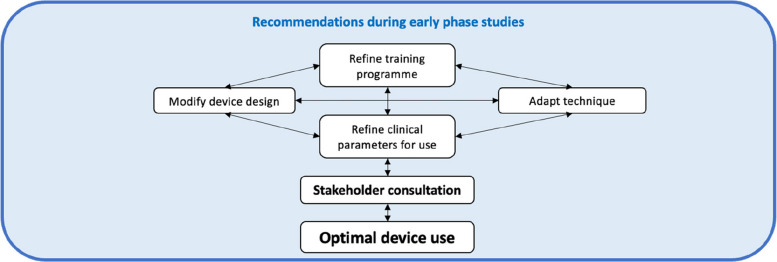
Framework for determining optimal device use through pre-clinical, protocol, and feasibility study considerations [29]

Following implementation of these key methodological considerations within ASSIST, we have proposed a framework for determining optimal device use during feasibility studies (Fig. 3).

The framework for determining optimal device use is a dynamic, iterative process that involves refining the training programme, modifying device design, adapting techniques, refining clinical parameters, and engaging in stakeholder consultations. These elements work together in a cyclical manner, each influencing the others, to ensure that the device is optimised for real-world use. The role of data collection is central to this process: feedback from pre-clinical studies, early-phase trials, case studies, and operator assessments continuously inform each element. Data helps refine training protocols, guide design modifications, adjust clinical techniques, and update clinical parameters to better align with real-world needs. By systematically collecting and analysing data at each stage, researchers can make evidence-based adjustments, ensuring that the device evolves in response to actual performance and user feedback, ultimately leading to the creation of a device that is both safe and effective for clinical use.

## Discussion

This paper aims to identify key methodological considerations relevant to the evaluation of innovative invasive devices in feasibility studies and propose a framework for their evaluation going forward. The challenges include optimising study design so that device use can be fully understood, particularly focussing on device design, technique, and clinical parameters for use as well as involving key stakeholders and industry partners throughout the journey. Consideration of these issues is recommended so that the appropriate study design and research questions can be formulated and so that operators can be correctly trained in the use of the device. Our experience demonstrates the importance of embedding qualitative research into the design of feasibility studies and how this methodology can be particularly crucial for the evaluation of complex innovative interventions. We gained many more insights into device use using this approach and gleaned these details much faster than just relying on the quantitative data.

Although qualitative case study methodology has previously been used to explore complex surgical interventions [30], there are no published examples of the use of these methods to investigate novel intrapartum devices. There was evidence that triangulation of data provided stakeholders with the ability to appraise findings in a quicker, more systematic way [8]. In our experience, the qualitative case study research allowed for constant feedback and discussion of the insight gained within the team as the study was progressing [29]. The manufacturers were able to develop and test new device design ideas based on our findings, and changes to the device use (technique) were quickly disseminated to all operators. Key updates included technique modifications like applying the device during contractions and adjusting the application angle, as well as design changes such as altering the deflation button and strengthening sleeve seals. These evidence-based changes were made to mitigate user errors and risks observed during testing. Feasibility work is essential to assess the use of an innovative invasive device in its intended clinical context, where there may be many variables, such as pain relief, positioning, and anatomy, that could influence the use and usability of the device. Furthermore, our IFU made explicit the clinical parameters for use (i.e. who/what/when/where should the device be used). In pre-clinical research, this information can be very sparse, as the device is still being developed and yet to be trialled in practice; clear documentation of changes to the eligibility and exclusion criteria need to be provided and justified. Updating the IFU requires the manufacturer and clinicians to work together as it is the manufacturer who must create the IFU but the operators who have the real-world device use experiences.

The operator training programme was iteratively updated, and new operators were always given the most up-to-date and accurate training programme, stemming directly from new data. This method of working (Fig. 2) allowed for optimal device use to be (i) determined efficiently, (ii) agreed upon by all stakeholders, (iii) data-driven and ultimately produced a safer device [29]. Challenges of undertaking case study methodology in this setting included the unpredictable nature of assisted vaginal births and the availability of the researcher. The volume of data collected was large, and it was at times challenging to present the complexities found in a simple way.

The engagement of stakeholders is fundamental to the advancement of research in AVB. While Patient and Public Involvement has become a standard requirement across research groups—shaping protocol development, study implementation, and dissemination—there are additional dimensions that warrant attention. For instance, the acceptability of participant observation and immediate postnatal interviews must be carefully considered to ensure meaningful data collection while respecting patient experiences and ethical boundaries.

Beyond participant engagement, early collaboration with ethics committees is crucial in AVB research. Proactively addressing ethical considerations during study design helps researchers navigate potential challenges, ensuring compliance with ethical frameworks and regulatory standards. This foresight strengthens the study’s integrity and facilitates smoother approval processes.

Furthermore, the intersection of clinical research and industry collaboration plays a pivotal role in driving innovation in AVB technologies. Aligning medical device development with real-world clinical challenges not only accelerates the translation of research into practical applications but also enhances patient safety and effectiveness. Strengthening these multidisciplinary partnerships is essential for the evolution of AVB practices, ensuring that emerging technologies are both evidence-based and clinically relevant.

## Conclusions

The development and evaluation of innovative medical devices, such as the OdonAssist, requires a multifaceted approach that integrates rigorous study design, stakeholder collaboration, and iterative feedback. Ensuring the safety, effectiveness, and usability of new devices is not solely a matter of quantitative outcomes but also benefits from qualitative methods to understand the complexities of device use in clinical settings. Embedding case study research within feasibility trials allows for rapid identification of issues and modifications that improve device performance and safety. Key to this process is the active involvement of stakeholders, including clinicians, industry partners, ethics committees, and patients, to ensure that the device meets real-world needs and is introduced safely into clinical practice. By fostering transparent communication, ongoing collaboration, and timely adaptation, researchers can optimise device design and technique, ultimately leading to improved patient care and outcomes.

Innovation is expected during the development of a medical device; however, it is vital that there are transparent and systematic methods surrounding the implementation of device modifications. We suggest that device design, technique for device use, clinical parameters for device use, and training in the use of the device are all intricately linked and need to be carefully considered in tandem. To do this, collaborative study design, execution, and monitoring is of paramount importance. Future research should continue to explore innovative methodologies to refine device development and enhance the understanding of device use in diverse clinical contexts.

## Data Availability

Not applicable.

## Abbreviation

IFU: Instructions for use
